# Aids to Nursing

**Published:** 1888-02-11

**Authors:** M. M. Basil

**Affiliations:** Author of "The Commoner Accidents to Life and Limb"


### Aids to Nursing.

By M. M. Basil, M.A., M.B., C.M., Author of "The Commoner Accidents to Life and Limb."

As a part of ventilation, you should encourage your patients to keep in the open air daily as long as possible. In no room are they likely to get air of such purity as out of the house. You will find that your very delicate patients, especially the so-called consumptive children, will, under proper precautions, benefit very largely by daily outing. Such of you as are on duty in the out-patients' department will observe that strumous children, with acute swellings in the neck, swollen tonsils, etc., come to us in larger numbers after a spell of cold or of rough weather. It is because they have been cooped up for days together like pilchards in a barrel, and disease is the result.

You cannot ventilate the sick-room in a house by itself, without attending at the same time to the ventilation of the whole house. Foul air will stream into your room from everywhere. It has been proved that no wall, however solid, is impervious to the passage of air. You can convince yourselves of this by looking any day to the ceiling of one of the wards that has not been recently whitewashed. You will notice that smoke, or carbon in a state of minute division, is deposited everywhere on the ceiling, but not uniformly. The smoky air has passed out through the structure and left the carbon behind it; but where the timber rafters are found, less air has permeated out and less carbon has been deposited; hence we have alternate strips of light and deep smoking of the roof. If the house is not ventilated, and you attempt to get one room in it well aired, that room becomes the aspirator of the entire house. It becomes the shaft through which all air-passages, corridors, every shut-up room, servant's room, lumber-room, sink, and lavatory in the house discharges its used-up air. It is, therefore, necessary that you should look after the ventilation of the whole system of connected rooms, especially as the medical attendant may not have the same means of observing every part of the house as you have. Should you notice anything very far wrong with any portion of the house, it would be well to inform him of it privately during the next visit.

Important as ventilation is in every case of disease, you must bear in mind that it is much more so in infectious diseases, in surgical cases, especially where there are recent wounds, and in cases of women after childbirth. In all cases of infection fresh air is our cheapest and most efficient disinfectant, and though many means of destroying infection are in vogue, and advantageously so, you cannot sufficiently bear in mind that they are all but broken reeds to rely upon. No inhabited room can be fumigated or disinfected efficiently. To ensure perfect fumigation we must of necessity vacate it very quickly. Minor degrees of fumigation have perhaps their advantage, inasmuch as they make ventilation compulsory. They are at best necessary nuisances, and the more fresh air you can secure the less disinfectants you will require. Perfuming a room is of course not ventilating or cleaning it, but only covering its foulness, though some perfumes are in a slight degree destroyers of infection.

Independently of its freshness or closeness, the dry or moist state of the air is also of importance in the sick-room; on this subject I shall give you one or two hints. In nearly all acute respiratory or lung diseases you must render the air moist. The justly-dreaded fear of the east wind in such diseases arises partly from the fact that it is a very dry wind. An observant nurse will at such times, by means of a bronchitis kettle, throw out steam into the room so as to secure the proper degree of moisture. In tracheotomy, and in wounds about the neck communicating with the windpipe, you must secure a very moist state of the atmosphere; in the last condition the first duty of the nurse would be to place a layer of moist muslin over the opening. In cases of phthisis without the complication of much bronchitis a dry state of the air is often well borne.

As ventilation and perfect cleanliness go hand in hand, you will easily understand that simply on the ground of having as little sewage in the air as possible, you must have no slop-pails in the room, no airing or drying of clothes, and no cooking. The sick-room must not be converted into a sewer. It should be made a sanctuary of cleanliness, and not a chamber of horrors.

As far as your own sick-room is concerned you must take the description given by Lord Tennyson as a highly "poetic" way of looking at the subject, arising simply from the poet's nose sniffing about in a fine frenzy, and finding that

> The air is damp and hush'd and close,
> As a sick man's room when he taketh repose,
> An hour before death.

The first and best test we possess as yet for ascertaining the state of freshness of a room is the well-trained nose in its unpoisoned condition. You must walk into the room from the outside air, and your first impressions of its freshness are the only reliable ones. After remaining in the room for a while the nose gets acclimatised to the place, the stuffy air acts as a narcotic poison to the nerve of smell, and then the nasal organ loses its delicate sense of perception. People differ much in their capability of judging of the freshness of air. Training improves the sense; knowledge of the importance of the subject sharpens the nose wonderfully, and living constantly in a pure atmosphere makes both the nose and the brain behind it very sensitive to impurities in the air. Those who are habitually poisoned get used to it, as it were; they become seasoned, though the so-called hardening process, here as almost in all cases, means a terrible loss in the end.

Secondly, a well-ventilated room or house should free itself of smells quickly. If there is proper circulation of air the smell should soon become imperceptible, unless it is continually generated. No house is properly ventilated if the smell of dinner, of burnt grease, paint, etc., sticks to it long.

Thirdly, you should have no yawning, gaping drowsiness, dulness, stupor, restlessness, irritability, languor, general malaise, headache, etc., in the ward. These are all appropriate to the church, the school-room, the theatre, the omnibus, and the railway carriage; and the sick ward should not encroach on the rights and privileges of any of these highly conservative institutions. If many of your patients begin to become irritable and "nasty" all round, you may generally depend upon it that, if the cook has not been recently changed, the windows and ventilators require attention. It would be interesting to determine how far the clergyman is answerable for the proverbially unattractive quality of sermons in general. Very often it is the brains of the congregation that must be pitied and blamed. No brain, when in a state of acute poisoning by foul air, is capable of judging any question impartially.

I shall say a few words next with regard to the choice of the sick-room, though the question is seldom referred to you, and when it is so referred it is really a case of "small choice in rotten apples." The question of disease is never taken into consideration in making our dwellings, although from the beginning it has been, and to the end of the chapter it will be the law, that no house can be inhabited long before questions of life and death must be decided in it. Though your endeavours to secure a model sick-room must often remain merely pious wishes, still you had better have some knowledge of its requirements. A good sick-room then, like a good bishop, must have many qualifications both positive and negative. It should be roomy, so as to afford ample original cubic footage, but not too large, so that it can be warmed and the perflation of air through it rendered possible. Some writers, and very recent ones too, demand that it should be "lofty," and so it should be if their idea of "loftiness" does not rise to more than twenty to twenty-five feet at the utmost. A room with a high ceiling is very unhealthy for the sick. Then it should be bright, warm, sunny; it should not have a north aspect, as the so-called "northern light," for which some make very material sacrifices, is a questionable boon in England. It should be away from noise, away from the laundry, the kitchen, the stable, sinks, and w.c. It should have a fireplace, and be fitted with windows reaching well up to the ceiling. You should especially get as far away as you possibly can from the w.c., remembering that one or two walls will not prevent the entrance of air from it into the sick-room, though apparently they may be situated well apart. In cases of operations, accidents, child-birth, and typhoid fever it would be a gross blunder to forget this point.

If the case is likely to be infectious, or if you are in doubt on the subject, it would be best to secure a good room as high as possible; the air from the sick-room would then get safely out of the house, and you would not be very liable to invasion by inquisitive visitors.

Few general rules can be laid down with regard to the furniture in the room. In many books on nursing you will meet with sweeping statemeuts which bear exaggeration, if not sweet innocence of the subject, in the very face of them. You should make the room as comfortable as possible. It should be as little different as you can make it from the usual arrangement of the patient's bed-room. If the case is a chronic or a non-infectious one no disturbance of carpets, furniture, drapery, etc., need be thought of. In acute diseases, especially if of an infectious nature, such as scarlet fever, measles, small-pox, typhoid fever, typhus fever, puerperal fever, diphtheria, erysipelas, pyæmia, etc., it would be best to allow no elaborate drapery, no woollen articles, no costly furniture, no carpets and valuable paintings. Some of these items, however, can be left in the room if the patient's means will allow of their being destroyed or efficiently disinfected.

Bed curtains, too, must be allowed or not according to the nature of each case. The sweeping assertion that they should be abolished will not stand the test of science or sense.

Screens also are useful articles for protecting the patient from draughts, the glare of light, heat, or during tidying of the room.

As a general rule the sick-room should have plenty of light; if possible, direct sunlight. Most convalescents and those suffering from chronic diseases bear sunlight well. But in many eye affections, in measles, small-pox, many acute brain diseases, in childbirth, and in cases where there is great irritability with exhaustion, the room must be darkened to a greater or lesser degree.

*(To be continued.)*

